# Supplementary material for: Establishment of a salt-induced bioremediation platform from marine Vibrio natriegens
Source: Commun Biol. 2022 Dec 9;5:1352. doi: 10.1038/s42003-022-04319-3 (PMC9734156; doi:10.1038/s42003-022-04319-3)
Supplement: Supplementary file 2 — Supplementary Information-New [file 42003_2022_4319_MOESM2_ESM.pdf]

Supplementary Information:

Establishment of a Salt-induced Bioremediation Platform from Marine *Vibrio natriegens*

Ling Huang[1], Jun Ni[1]*, Chao Zhong[2], Ping Xu[1], Junbiao Dai[2], and Hongzhi Tang[1]*

[1]State Key Laboratory of Microbial Metabolism, and School of Life Sciences & Biotechnology, Shanghai Jiao Tong University, Shanghai 200240, People's Republic of China

[2]CAS Key Laboratory of Quantitative Engineering Biology, Guangdong Provincial Key Laboratory of Synthetic Genomics and Shenzhen Key Laboratory of Synthetic Genomics, Shenzhen Institute of Synthetic Biology, Shenzhen Institutes of Advanced Technology, Chinese Academy of Sciences, Shenzhen, 518055, P.R. China

*Corresponding author: H. Z. Tang or J. Ni

Mailing address: School of Life Sciences & Biotechnology, Shanghai Jiao Tong University, Shanghai 200240, P. R. China

E-mail: tanghongzhi@sjtu.edu.cn or tearroad@163.com; Tel: +86-21-34204066; Fax: +86-21-34206723

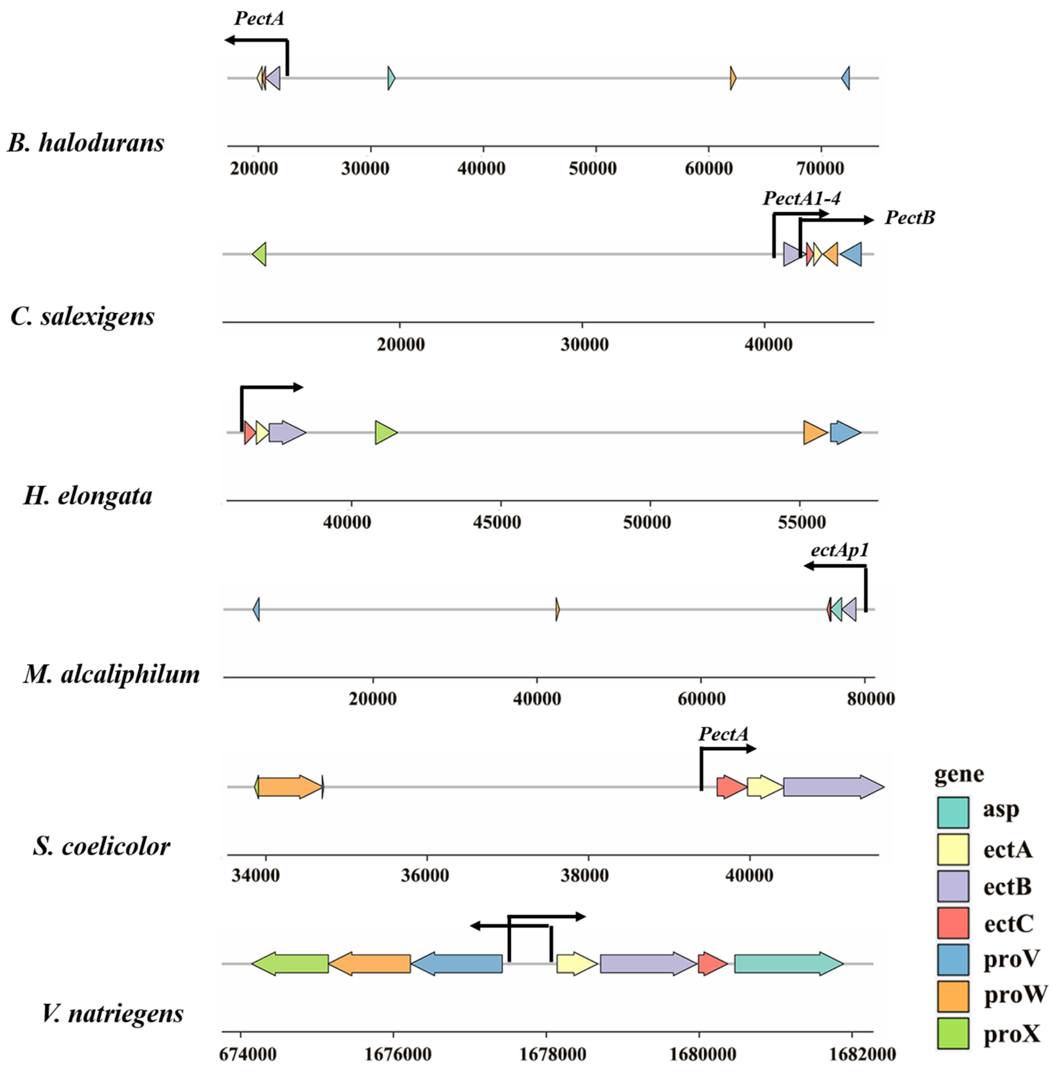

Supplementary Figure 1. The ectoine biosynthesis genes and glycine betaine/proline transport system genes' distribution in the most researched halophilic and halotolerance microorganisms: *Methylotuvimicrobium alcaliphilum* 20Z (NC_016112.1)[36], *Bacillus halodurans* C-125 (BA000004.3)[37], *Streptomyces coelicolor* A3 (AL645882.2)[38], *Halomonas elongata* strain HEK1 (FN869568.2)[39], and *Chromohalobacter salexigens* DSM 3043 (CP000285.1)[40].

Supplementary Table 1 Primers used for activity determination of the promoters

Primer names	Sequences	Function
BBa-P1F	attcgcggccgcatctagaggatgttattacgacggactt	
BBa-P1R	ttctcctctttcactagtacagctggaaccaagatcaca	Construction of pSK8k-P1-mRFP
BBa-P2F	attcgcggccgcatctagag catatgatgtcgtcaccgtc	
BBa-P2R	ttctcctctttcacta ttggttgattagacagt	Construction of pSK8k-P2-mRFP
BBaF	tactagtgaaagaggagaa	
BBaR	ctctagatgcggccgcg	Construction of pSK8k-P1/P2-mRFP
BBa-p12R	ttctcctctttcacta catatgatgtcgtcaccgtc	Construction of pSK8k-P12-mRFP
BBa-p21R	ttctcctctttcacta gatgttattacgacggactt	Construction of pSK8k-P21-mRFP
BBaGR-F	cctagttcgttgatattttttcaaa	Construction of pSK8k-P12-2-mRFP
BBaGR-R	ctaggatggcttcctccg	Construction of pSK8k-P12-2-mRFP
0127-BBaR	cagaaatcatccttag	Construction of pSK8k-P12-2-mRFP
0127-BBaFP1	gatgttattacgacggactt	Construction of pSK8k-P1-Mrfp-eGFP
0127-BBaFP2	catatgatgtcgtcaccgtc	Construction of pSK8k-P2-Mrfp-eGFP
GFPF	ctaaggatgatttctg ttacttgtacagctcgtcca	Construction of pSK8k-P1/P2-Mrfp-eGFP
GFPRP12	gtcgtaataacatcaagcttatggtgagcaagggcgagga	Construction of pSK8k-P12-Mrfp-eGFP
GFPRP21	gacggtgacgacatcatatgatggtgagcaagggcgagga	Construction of pSK8k-P21-Mrfp-eGFP

Supplementary Table 2 Primers used for construction of degrading models

Primer names	Sequences	Function
PAMp12mrF	ccggaattcttatttcgggttcaccacgc	Construction of pAM-mpd-p12
PAMP12mrR	aagtccgtcgtaataacatcatgccgctgaaaaaccgcct	
PAMP12F	gatgttattacgacggactt	Construction of pAM-mpd-p12
PAMP12R	catatgatgtcgtcaccgtc	
PAMP12tijF	gacggtgacgacatcatatgatgatccgcaccggcaaaca	Construction of pAM-mpd-p12-xtij
PAMP12tijR	ccgctcgagttagctgccagcgcgagcag	
PAMP12R-xhoi	ccgctcgagcatatgatgtcgtcaccgtc	Construction of pAM-p12
168A1EcorI-RpAM	cacaggaaacagaccatggaattcctactcgcaggtcttc	Construction of pAM-168A1p12
168A1p12XhoI-R	gatcctttaccagactcgaggcggcgagttcgccattgag	
168A1FdFNRXhoI-R	gatcctttaccagactcgagggtacctcacttctcgacga	Construction of pAM-168A1p12FdFNR
TcpX-F	ttcacacaggaaacagaccatgtcttctgcggtttctatc	Construction of pAM-mpd-p12-xtij
TCPX-R	ttgccggtgcggatcatagaaccaccaccaccatccgcaactgggtgagt	
PAMPTRC-F	ggtctgtttcctgtgtgaa	Construction of pAM-mpd-p12-xtij
PAMPTRC-R	atgatccgcaccggcaaaca	
P12TCPX-F	ggtgacgacatcatatgatgtcttctgcggtttctatcga	Construction of pAM-mpd-p12-xtij
PAMP12-F	atgatgtcgtcaccgtcaga	
PETaseF1	tgatttctggaattcttaatggtgatggtgatgatgagaacagttagcagt	Construction of pSK8k-PPM
PETaseR1	ataacatcaagcttaatgcaaactaacccg	
MHETaseF1	atatcaacgaactagatgcaaactactgttactac	Construction of pSK8k-PPM
MHETaseR1	gcactatcagcgttattaatggtgatggtgatgatgcggcggagcagcac	
LccF1	tgatttctggaattcttaatggtgatggtgatgatgttgacagtgacggtt	Construction of pSK8k-LPT
LccR1	ataacatcaagcttaatggacggtgttctg	
TfcaF1	atatcaacgaactaggtggaaatcgttatc	

TfcaR1	gcactatcagcgttattaatggtgatggtgatgatgcagcggaacaccgt	
MHETaseF2	tgatttctggaattcttaatggtgatggtgatgatgcggcggagcagcaca	Construction of pSK8k-MPP
MHETaseR2	ataacatcaagcttaatgcaaactactgtt	
PETaseF2	atatcaacgaactagatgcaaactaacccg	Construction of pSK8k-MPP
PETaseR2	gcactatcagcgttattaatggtgatggtgatgatgagaacagttagcag	
TfcaF2	tgatttctggaattcttaatggtgatggtgatgatgcagcggaacaccgtc	Construction of pSK8k-TPL
TfcaR2	ataacatcaagcttagtggaaatcgttatc	
LccF2	atatcaacgaactagatggacggtgttctg	Construction of pSK8k- TPL
LccR2	gcactatcagcgttattaatggtgatggtgatgatgttgacagtgacggt	
pet-BBA-F	taacgctgatagtgctagtg	Construction of pSK8k-pet hydrolases
pet-BBA-R	gaattccagaaatcatcctt	

Supplementary Table 3 **Summary of *Vmax* genes with high transcription levels under high salt concentration (5% NaCl) (up-regulation fold > 4)**

log2 (X5/X1)	Name for shorten	Nr
9.468815759	ectA	WP_020336357.1|1.8e-98|diaminobutyrate acetyltransferase
9.194209516	tctC	WP_014231940.1|5.4e-181|MULTISPECIES: tripartite tricarboxylate transporter substrate-binding protein
9.020519552	proW	WP_065302445.1|5.2e-201|proline/glycine betaine ABC transporter permease ProW
8.968519569	ectB	WP_065302446.1|2.6e-244|diaminobutyrate--2-oxoglutarate transaminase
8.876979793	lysC	WP_065302447.1|3.5e-269|aspartate kinase
8.861094531	proX	WP_065302444.1|1.2e-194|proline/glycine betaine ABC transporter substrate-binding protein ProX
8.787629164	proV	WP_065299720.1|2.1e-110|proline/glycine betaine ABC transporter ATP-binding protein ProV
8.515744031	ectC	WP_014231962.1|2.2e-69|MULTISPECIES: ectoine synthase
7.532546721	csiD	WP_014233866.1|4.2e-186|MULTISPECIES: carbon starvation induced protein
7.406908107	lhgO	WP_014233865.1|1.8e-242|MULTISPECIES: L-2-hydroxyglutarate oxidase
7.162683407	fliC	WP_020333613.1|2.9e-202|flagellin
6.842980096	tctA	WP_014231942.1|1.4e-279|MULTISPECIES: tripartite tricarboxylate transporter permease
6.802780661		WP_065303233.1|4.9e-190|porin
6.557296558	tctB	WP_020336343.1|2.3e-87|tripartite tricarboxylate transporter TctB family protein

6.199531497	TC.BCT	WP_065303395.1|0.0e+00|transporter
6.094543291	TC.BCT	WP_020335534.1|7.1e-300|BCCT family transporter
5.942619538		WP_020334225.1|3.9e-168|TRAP transporter substrate-binding protein
5.664750648	phbC, phaC	WP_014233788.1|0.0e+00|MULTISPECIES: class I poly(R)-hydroxyalkanoic acid synthase
5.663912479	ompW	WP_065303206.1|2.3e-119|outer membrane protein OmpW
5.655412213		WP_065303388.1|1.7e-193|ornithine cyclodeaminase
5.593320831		WP_020335107.1|0.0e+00|molybdopterin guanine dinucleotide-containing S/N-oxide reductase
5.590975025	FlaG	WP_014232677.1|1.2e-71|MULTISPECIES: flagellar protein FlaG
5.570136946	mcp	WP_014234664.1|0.0e+00|methyl-accepting chemotaxis protein
5.560438547		WP_014233787.1|3.6e-55|MULTISPECIES: phasin family protein
5.435044413	Fla	WP_014232679.1|4.0e-204|MULTISPECIES: flagellin
5.382086212	phoE	WP_065302149.1|4.8e-185|porin
5.349578955	TC.BCT	WP_020333247.1|1.5e-305|BCCT family transporter
5.186841808	aldB	WP_079402443.1|1.0e-279|aldehyde dehydrogenase
5.136134692		WP_020335700.1|1.2e-80|hypothetical protein
5.109897321		WP_014233250.1|6.4e-44|MULTISPECIES: DUF4212 domain-containing protein
4.947617395	NhaC	WP_014233857.1|2.7e-269|Na^+/H^+ antiporter NhaC

4.853986711		WP_014233785.1|1.4e-133|MULTISPECIES: beta-ketoacyl-ACP reductase
4.741901323		WP_024372614.1|2.1e-213|MULTISPECIES: cytochrome c
4.72703998		WP_065303360.1|8.4e-293|methyl-accepting chemotaxis protein
4.703397239		EPM41066.1|5.6e-220|3-ketoacyl-CoA thiolase
4.643806603		WP_020333703.1|1.1e-133|flagellar motor protein PomA
4.614912285		WP_020336182.1|3.1e-113|sel1 repeat family protein
4.514735365		WP_065303463.1|0.0e+00|choline dehydrogenase
4.490939974		WP_014233750.1|8.8e-119|MULTISPECIES: 3,4-dihydroxy-2-butanone-4-phosphate synthase
4.40654012		WP_020333793.1|2.9e-279|betaine-aldehyde dehydrogenase
4.380762037	MotB	WP_014231020.1|1.5e-175|MULTISPECIES: flagellar motor protein MotB
4.269675623		WP_065303118.1|4.1e-223|sel1 repeat family protein
4.243913451	Fla	WP_065296319.1|4.8e-205|flagellin
4.183060922		WP_014230768.1|8.1e-222|MULTISPECIES: methyl-accepting chemotaxis protein
4.152015077		WP_014233980.1|3.5e-106|MULTISPECIES: transcriptional regulator BetI
4.147621822		WP_014234152.1|1.9e-59|MULTISPECIES: RidA family protein
4.144582585		WP_065303633.1|1.1e-259|methyl-accepting chemotaxis protein
4.110199267		WP_065303552.1|1.5e-280|pyridine nucleotide-disulfide oxidoreductase

4.070032566	FliS	WP_020333610.1|9.9e-68|flagella export chaperone FliS
4.068173534		WP_065302219.1|0.0e+00|hypothetical protein
4.018023497		WP_065297217.1|2.4e-27|hypothetical protein
4.007473577		ANQ19339.1|1.8e-50|sarcosine oxidase
