# Supplementary material for: Establishment of a salt-induced bioremediation platform from marine Vibrio natriegens
Source: Commun Biol. 2022 Dec 9;5:1352. doi: 10.1038/s42003-022-04319-3 (PMC9734156; doi:10.1038/s42003-022-04319-3)
Supplement: Supplementary file 3 — Reporting Summary-New [file 42003_2022_4319_MOESM3_ESM.pdf]

nature portfolio

Corresponding author(s): Hongzhi Tang
Last updated by author(s): Nov 21, 2022

Reporting Summary

Nature Portfolio wishes to improve the reproducibility of the work that we publish. This form provides structure for consistency and transparency in reporting. For further information on Nature Portfolio policies, see our Editorial Policies and the Editorial Policy Checklist.

Statistics

For all statistical analyses, confirm that the following items are present in the figure legend, table legend, main text, or Methods section.

n/a	Confirmed	
☐	☒	The exact sample size (*n*) for each experimental group/condition, given as a discrete number and unit of measurement
☐	☒	A statement on whether measurements were taken from distinct samples or whether the same sample was measured repeatedly
☐	☒	The statistical test(s) used AND whether they are one- or two-sided<br>*Only common tests should be described solely by name; describe more complex techniques in the Methods section.*
☐	☒	A description of all covariates tested
☐	☒	A description of any assumptions or corrections, such as tests of normality and adjustment for multiple comparisons
☐	☒	A full description of the statistical parameters including central tendency (e.g. means) or other basic estimates (e.g. regression coefficient) AND variation (e.g. standard deviation) or associated estimates of uncertainty (e.g. confidence intervals)
☐	☒	For null hypothesis testing, the test statistic (e.g. *F*, *t*, *r*) with confidence intervals, effect sizes, degrees of freedom and *P* value noted<br>*Give P values as exact values whenever suitable.*
☒	☐	For Bayesian analysis, information on the choice of priors and Markov chain Monte Carlo settings
☐	☒	For hierarchical and complex designs, identification of the appropriate level for tests and full reporting of outcomes
☐	☒	Estimates of effect sizes (e.g. Cohen's *d*, Pearson's *r*), indicating how they were calculated

Our web collection on statistics for biologists contains articles on many of the points above.

Software and code

Policy information about availability of computer code

Data collection	*Provide a description of all commercial, open source and custom code used to collect the data in this study, specifying the version used OR state that no software was used.*
Data analysis	*Provide a description of all commercial, open source and custom code used to analyse the data in this study, specifying the version used OR state that no software was used.*

For manuscripts utilizing custom algorithms or software that are central to the research but not yet described in published literature, software must be made available to editors and reviewers. We strongly encourage code deposition in a community repository (e.g. GitHub). See the Nature Portfolio guidelines for submitting code & software for further information.

Data

Policy information about availability of data

All manuscripts must include a data availability statement. This statement should provide the following information, where applicable:

- Accession codes, unique identifiers, or web links for publicly available datasets
- A description of any restrictions on data availability
- For clinical datasets or third party data, please ensure that the statement adheres to our policy

All data supporting the findings of this study are available within the article and

its Supplementary Information file (Supplementary Figure 1, and Supplementary Table 1-3). Additional information, relevant data and unique biological materials will be available from the corresponding author upon reasonable request.

Human research participants

Policy information about studies involving human research participants and Sex and Gender in Research.

Reporting on sex and gender	*Use the terms sex (biological attribute) and gender (shaped by social and cultural circumstances) carefully in order to avoid confusing both terms. Indicate if findings apply to only one sex or gender; describe whether sex and gender were considered in study design whether sex and/or gender was determined based on self-reporting or assigned and methods used. Provide in the source data disaggregated sex and gender data where this information has been collected, and consent has been obtained for sharing of individual-level data; provide overall numbers in this Reporting Summary. Please state if this information has not been collected. Report sex- and gender-based analyses where performed, justify reasons for lack of sex- and gender-based analysis.*
Population characteristics	*Describe the covariate-relevant population characteristics of the human research participants (e.g. age, genotypic information, past and current diagnosis and treatment categories). If you filled out the behavioural & social sciences study design questions and have nothing to add here, write "See above."*
Recruitment	*Describe how participants were recruited. Outline any potential self-selection bias or other biases that may be present and how these are likely to impact results.*
Ethics oversight	*Identify the organization(s) that approved the study protocol.*

Note that full information on the approval of the study protocol must also be provided in the manuscript.

Field-specific reporting

Please select the one below that is the best fit for your research. If you are not sure, read the appropriate sections before making your selection.

☐ Life sciences ☐ Behavioural & social sciences ☒ Ecological, evolutionary & environmental sciences

For a reference copy of the document with all sections, see nature.com/documents/nr-reporting-summary-flat.pdf

Ecological, evolutionary & environmental sciences study design

All studies must disclose on these points even when the disclosure is negative.

Study description	Salt-induced promoters are identified in Vibrio natriegens and used to engineer a strain for potential application in degradation of microplastics in marine water.
Research sample	One-gram PET (containing 0.5 g micro-PET, and 0.5 g PET membrane), 100 mg/L CP, or 1 mg/L HBCDs were added to 20 mL of the cell lysate as the substrate, separately, all the reactions were carried out at 30℃.
Sampling strategy	One mL reaction mixture was extracted from the reaction system every 2 days. Hydrochloric acid (1%) was added to samples to terminate the reaction. All samples were stored at -80℃ until use, and three biologically independent samples were detected for each single point
Data collection	The concentration and intermediate metabolites of CP were detected by gas chromatography-mass spectrometry (GC-MS) (Agilent & GC-7890B; MS-5977B) detection. The organic phase was incubated with an equal volume of BSTFA at 70℃ for 30 min before injection. HBCDs were quantified by ultra-performance liquid chromatography-quadrupole time-of-flight mass spectrometry (UPLC-TOF/MS) equipped with an Eclipse XDB C18 analytical column (5 µm, 4.6 × 150 µm, Keystone Scientific, Agilent). A mobile phase of water and methanol at a flow rate of 0.25 mL/min was applied for the target compounds. The proportional gradient of the mobile phase was started at 95% methanol, and increased linearly to 100% over 25 min, then decreased immediately to 95% and held for 10 min. For mass spectrometry analysis, the ionization source was run in negative mode, and MS detection was set from m/z 0 to 1,700.Bis-(2-hydroxyethyl) terephthalic acid (BHET) and mono-(2-hydroxyethyl) terephthalic acid (MHET) were quantified by ultra-performance liquid chromatography (HPLC) equipped with an Eclipse XDB C18 analytical column (5 µm, 4.6 × 150 µm, Keystone Scientific, Agilent). A mobile phase of water with 0.1% formic acid and methanol at a flow rate of 0.8 mL/min was applied for the target compounds. The proportional gradient of the mobile phase was started at 5% methanol, and increased linearly to 44% over 12 min, then increased linearly to 70% over 3 min and held for an additional 3 min before returning to 5% methanol immediately.
Timing and spatial scale	One mL reaction mixture was extracted from the reaction system every 2 days for To determine their ability to degrade PET, CP, or HBCDs, the engineered strains Vmax-mpdp12tcpXA, Vmax-cyp168A1p12FdFNR, Vmax-PETaseP122MHETase (PPM), Vmax-MHETaseP122PETase (MPP), Vmax-LCCP122Tfca (LPT), and Vmax-TfcaP122LCC (TPL). ;1 mL reaction mixture was extracted from the reaction system every 2 h, for the reaction with PPM, MPP, LPT, and TPL constructs' crude enzyme.
Data exclusions	Concentration-degradation-curves were generated with GraphPad Prism 8.0 software.

Reproducibility	All assays were performed in duplicates and repeated in least three independent experiments.
Randomization	All assays were performed in duplicates and repeated in least three independent experiments.
Blinding	All assays were performed in duplicates and repeated in least three independent experiments.

Did the study involve field work? ☐ Yes ☒ No

Reporting for specific materials, systems and methods

We require information from authors about some types of materials, experimental systems and methods used in many studies. Here, indicate whether each material, system or method listed is relevant to your study. If you are not sure if a list item applies to your research, read the appropriate section before selecting a response.

Materials & experimental systems

n/a	Involved in the study
☒	☐ Antibodies
☒	☐ Eukaryotic cell lines
☒	☐ Palaeontology and archaeology
☒	☐ Animals and other organisms
☒	☐ Clinical data
☒	☐ Dual use research of concern

Methods

n/a	Involved in the study
☒	☐ ChIP-seq
☒	☐ Flow cytometry
☒	☐ MRI-based neuroimaging
